# Supplementary material for: A SARS-CoV-2 neutralizing antibody with extensive Spike binding coverage and modified for optimal therapeutic outcomes
Source: Nat Commun. 2021 May 11;12:2623. doi: 10.1038/s41467-021-22926-2 (PMC8113581; doi:10.1038/s41467-021-22926-2)
Supplement: Supplementary file 1 — Supplementary Information [file 41467_2021_22926_MOESM1_ESM.pdf]

Supplementary information for

A SARS-CoV-2 neutralizing antibody with extensive Spike binding coverage and modified for optimal therapeutic outcomes

Guo et al.

Supplementary Figures 1-10

Supplementary Tables 1-4

Supplementary Figure 1

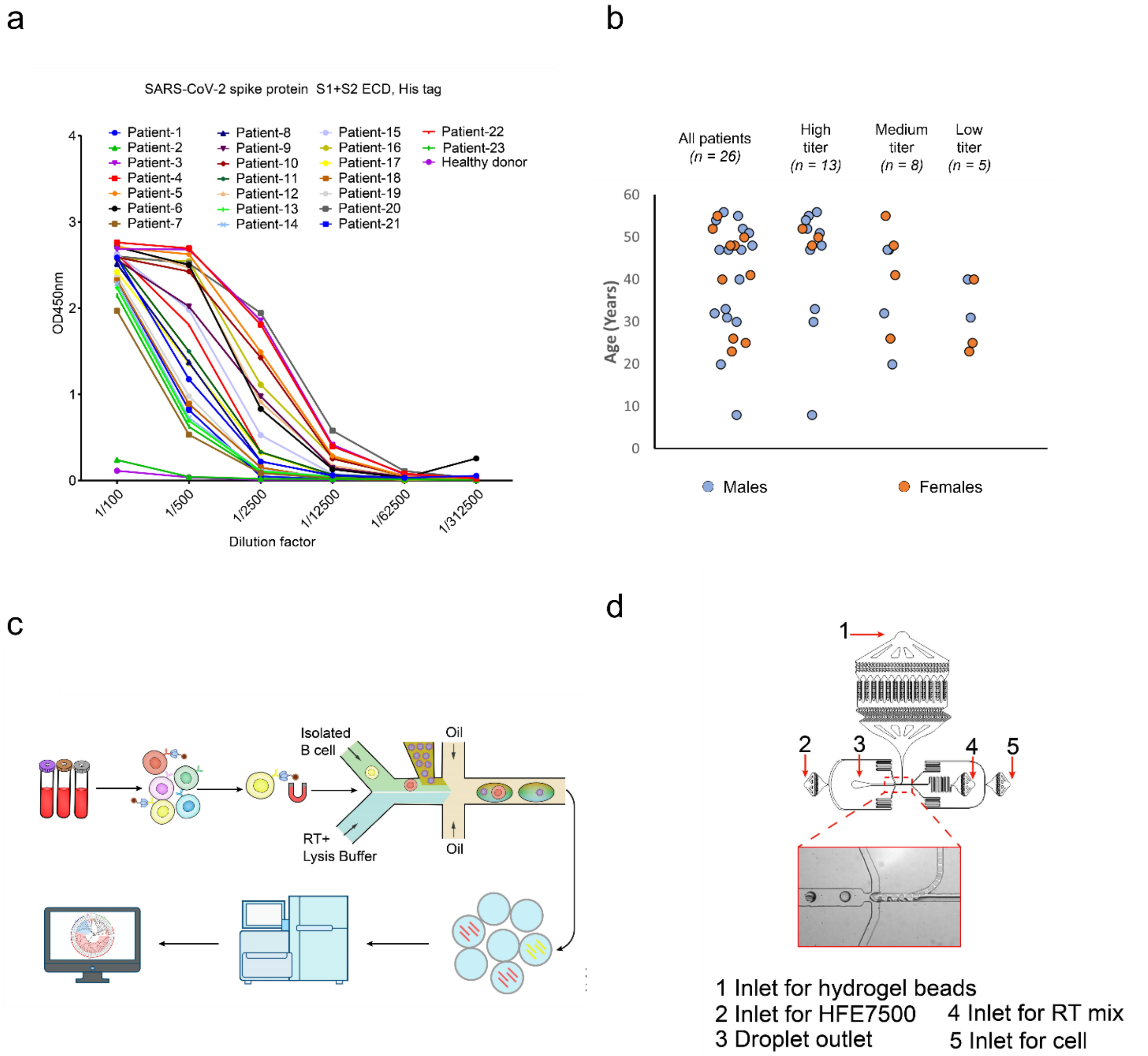

Supplementary Fig.1. Analyses of antibody responses to SARS-CoV-2 proteins and antibody identification from convalescent patients using single B cell sequencing. (a) Serums from 23 convalescent patients and one healthy donor were analyzed for their binding abilities to the SARS-CoV-2 spike protein using ELISA. Samples at different dilutions were tested in duplicates and mean is shown. (b) Classification of patient samples into high (>2500), medium (500-2500), and low (<500) titer categories. (c) Schematic diagram of the antibody identification from convalescent patients using single B cell sequencing. SARS-CoV-2 S protein binding B cells were isolated from PBMC of convalescent patients with magnetic beads that conjugated with biotinylated S protein as probes. The isolated cells were individually co-compartmentalized in droplets along with lysis buffer, reverse transcriptase and one hydrogel bead. Each hydrogel bead

carried VH and VL specific oligos tagged with a unique barcode. The resulting cDNAs from one cell carried an identical barcode. The barcoded cDNAs were sequenced to identify cognate heavy and light chain pairs. (d) The design of the microfluidics chip for co-compartmentalization of single cells and single hydrogel bead in droplets.

Supplementary Figure 2

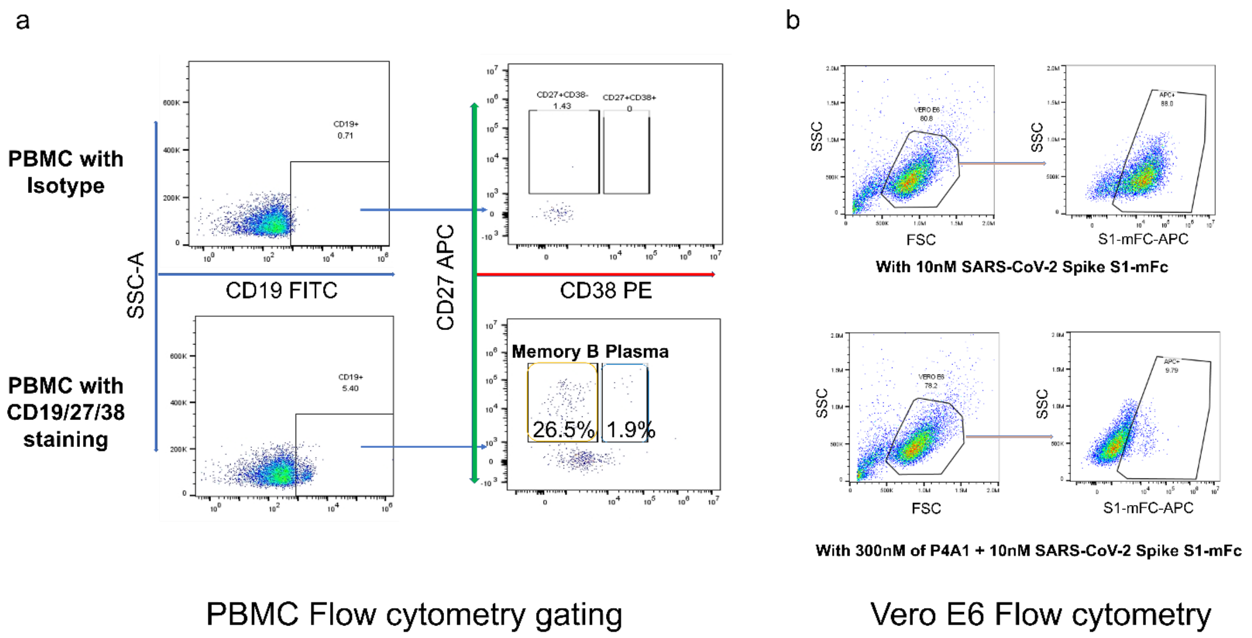

Supplementary Fig. 2. Gating strategy for patient B cell analysis and Vero E6 cells. (a) Gating strategy for patient B cell analysis. B cells: $CD19^{+}$; Memory B cells: $CD19^{+}CD27^{+}CD38^{-}$; Plasmablasts: $CD19^{+}CD27^{+}CD38^{+}$. (b) Gating strategy for antibody blocking of S1 binding to Vero E6 cells. Live Vero E6 cells were gated by FSC/SSC, then S1-mFc binding to Vero E6 cells were visualized by positive anti-mFc-AF647 staining.

Supplementary Figure 3

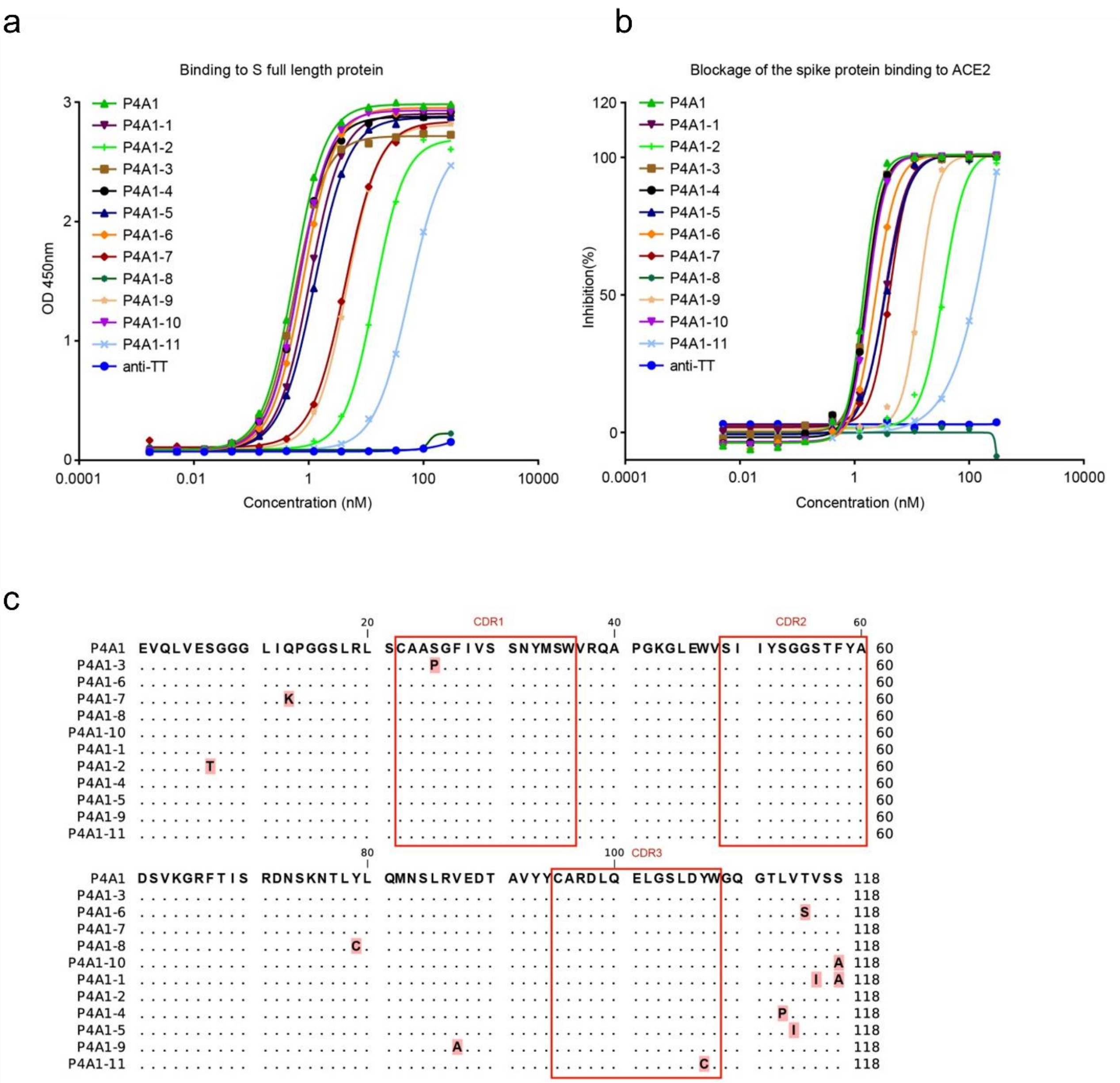

Supplementary Fig.3. Identification and characterization of somatic variants of antibody P4A1. Eleven heavy chain sequences closely related to P4A1 were bioinformatically identified from NGS results. These P4A1-class heavy chains were reconstituted with the light chain from P4A1 and numbered P4A1-1 to P4A1-11. (a) Binding of the antibodies to the full-length S protein was evaluated by ELISA. (b) Blocking the binding of S1 protein to Vero E6 cell line by antibodies was evaluated by flow cytometry. (a) and (b), antibodies at different dilutions were tested in duplicates and mean is shown. (c) The amino acid sequences of the P4A1-class antibodies were aligned.

Supplementary Figure 4

a

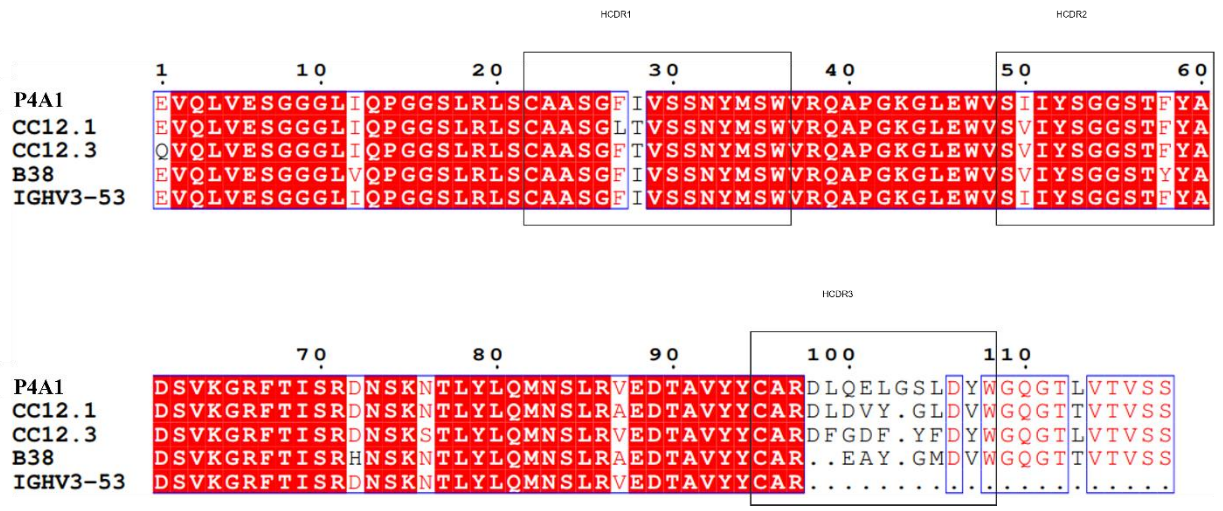

b

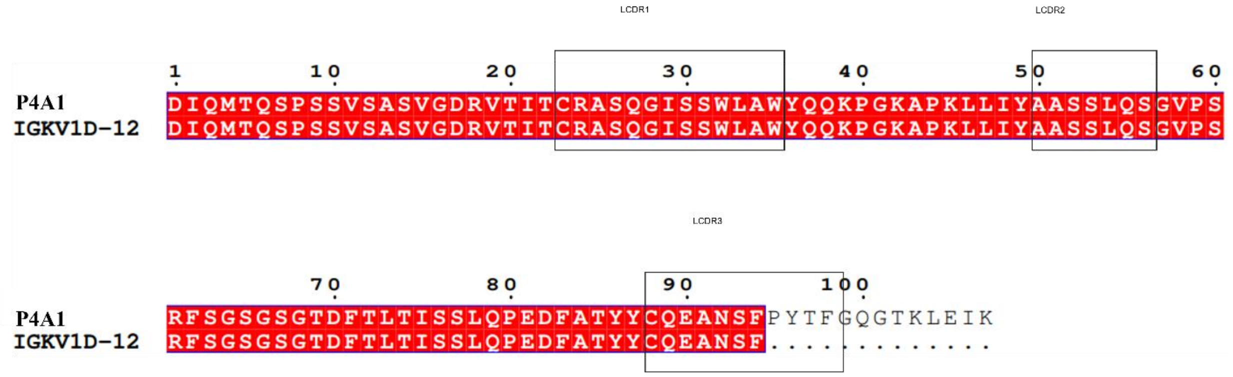

c

Antibody	Heavy chain	Light chain
P4A1	IGHV3-53	IGKV1-12
CC12.1	IGHV3-53	IGKV1-9
CC12.3	IGHV3-53	IGKV3-20
B38	IGHV3-53	IGKV1-9

Supplementary Fig.4. Sequence alignment of P4A1 to other reported antibodies from the IGHV3-53 germline. (a) Alignment of the heavy chain variable domain sequence of P4A1 with CC12.1, CC12.3, B38 and the germline IGHV3-53 sequence. (b) Alignment of the light chain variable domain sequence of P4A1 with the germline IGKV1D-12 sequence. (c) Germline usage comparison of heavy chain and light chain discussed in this paper.

Supplementary Figure 5

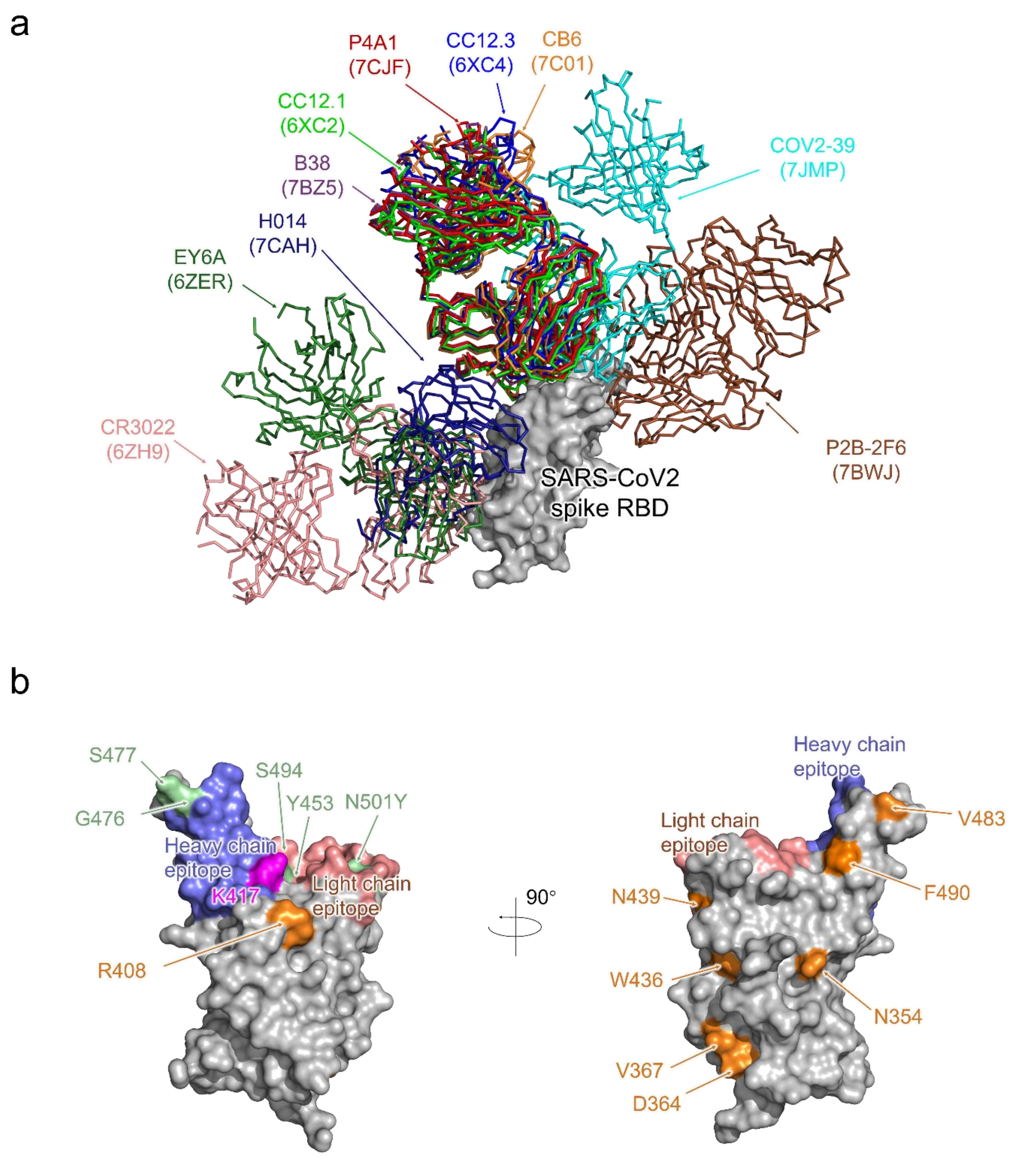

Supplementary Fig.5. Structural comparison of the binding mode among P4A1 and several reported RBD-specific neutralizing antibodies from various germlines. (a) Superposition of P4A1 (red, PDB 7CJF), CC12.1 (green, PDB 6XC2), CC12.3 (blue, PDB 6XC4), B38 (purple, PDB 7BZ5), CB6 (orange, PDB 7C01), COV2-39 (cyan, PDB 7JMP), H014 (deep blue, PDB 7CAH), EY6A (forest green, PDB 6ZER), CR3022 (salmon red, PDB 6ZH9), P2B-2F6 (brown, PDB 7BWJ), to SARS-CoV-2 spike glycoprotein RBD (gray). (b) Surface representation of several clinical isolates

with Spike RBD mutations. The SARS-CoV-2 RBD is colored in gray and displayed in surface representation. The epitope of P4A1 heavy chain (slate blue), light chain (salmon red), residue K417 (pink) are displayed and colored as Figure2. The clinic mutations Y453, G476, S477, S494, and N501Y, which located at the edge of the P4A1 epitope are colored in pale green. The clinic mutations N354, D364, V367, R408, W436, N439, V483, and F490, which are adjacent to the epitope residues or on the opposite side of the P4A1 epitope, are colored in orange.

Supplementary Figure 6

a

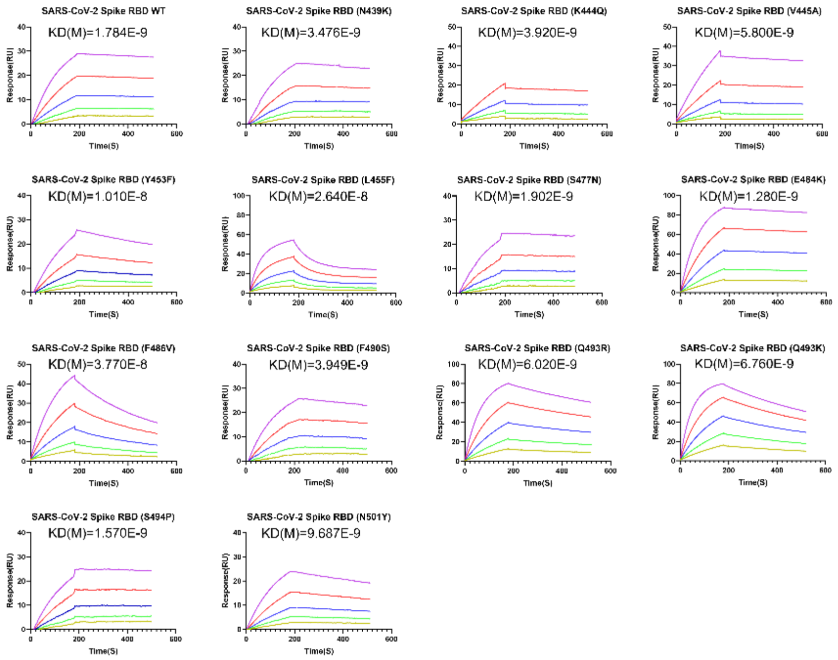

b

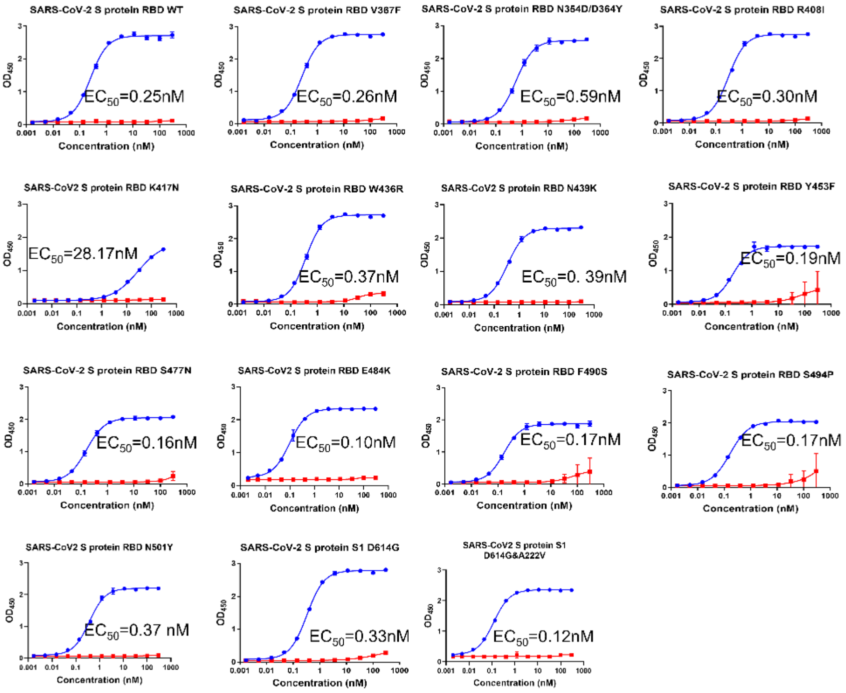

Supplementary Fig. 6. The binding of P4A1-2A. (a) Binding affinity to WT or RBD mutants by SPR. (b) Binding of P4A1-2A (blue) or isotype control (red) antibodies to the SARS-CoV-2 S

protein RBD or S1 variants was determined by ELISA. The 384-well plates were coated with 20 nM of the respective SARS-CoV-2 S protein RBD/S1 variants. The binding of P4A1-2A or isotype control antibodies (12 concentrations obtained by 3-fold serial dilutions of a 300 nM antibody stock solution, in triplicate) was detected by goat $F(ab')_2$ anti-human IgG (H+L)-HRP. Data are presented as mean ± SD. Most of the experiments were repeated once or not repeated because data from SPR or ELISA assays are supported by each other.

Supplementary Figure 7

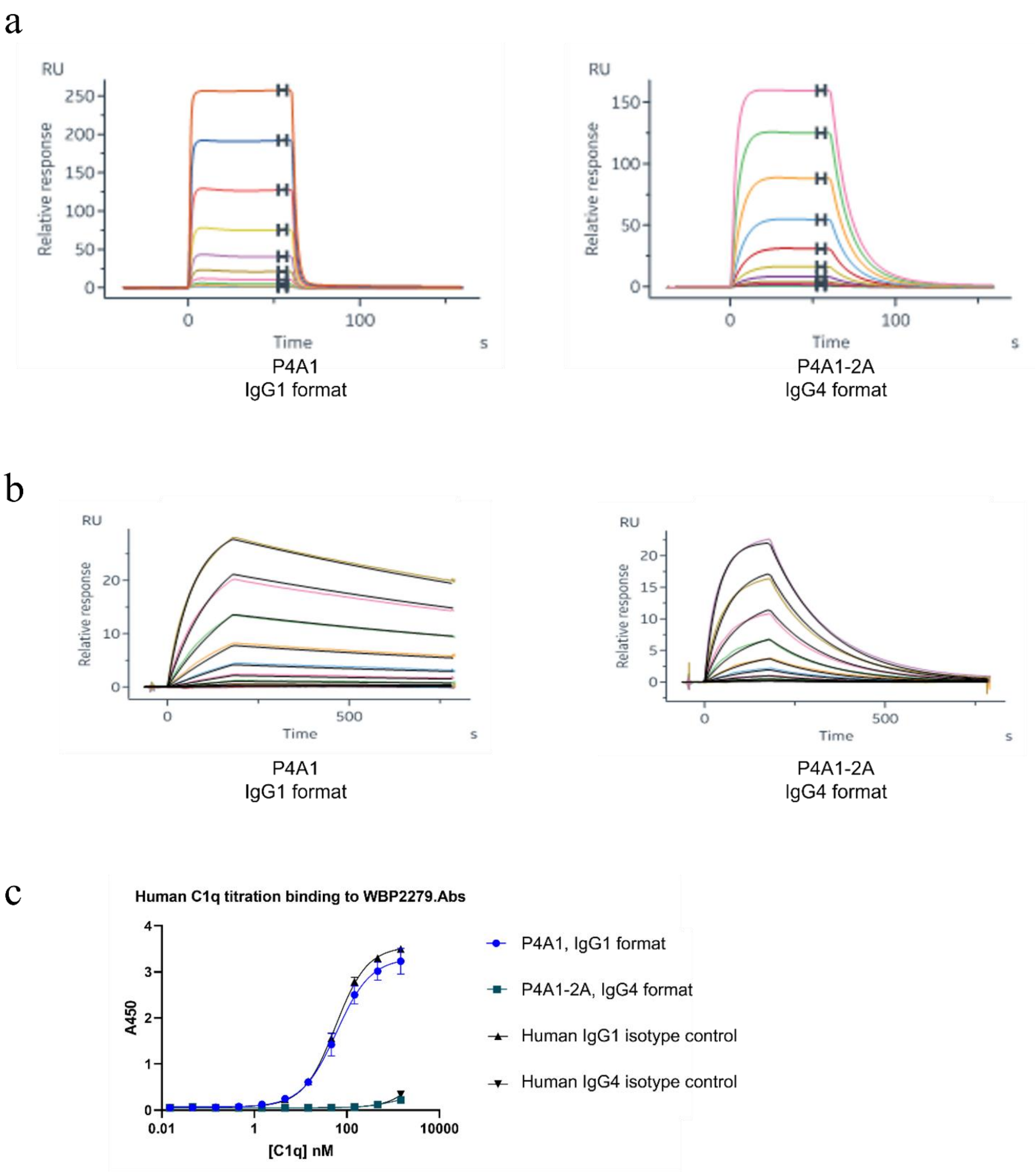

Supplementary Fig.7. Binding of P4A1 (IgG1 form) and P4A1-2A (IgG4 form) to FcRn (a), FcγRI (b) by SPR using Biacore 8K and C1q (c) by ELISA. Assays are detailed in material and methods. (c) C1q at different concentrations were tested in triplicates. Data are presented as mean ± SD. Experiments were not repeated as controls were consistent with historical data and/or literature.

Supplementary Figure 8

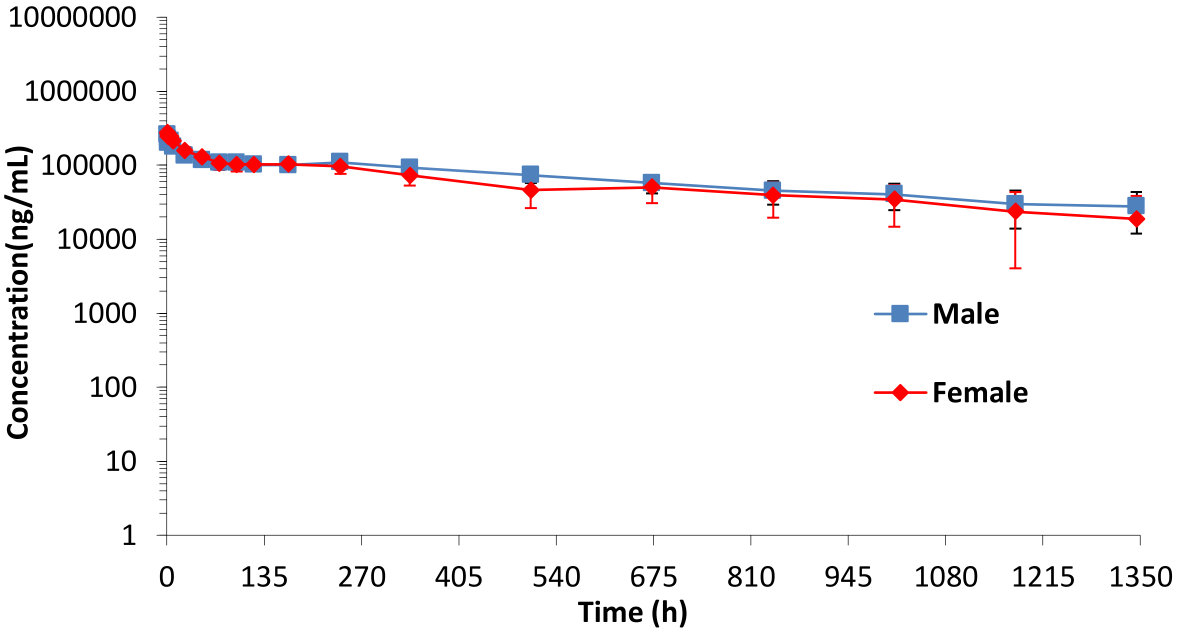

**PK Parameter**	**Mean (Male)**	**Mean (Female)**
C_{max} (ng/mL)	259000	273000
C_{last} (ng/mL)	27700	13000
T_{max} (h)	1.0833	1.0833
$T_{1/2}$ (h)	586	332
$AUC_{0\text{-}last}$ (ng.h/mL)	88200000	66700000

Supplementary Fig.8. Mean serum P4A1-2A concentration in cynomolgus monkeys. Male and female cynomolgus monkeys (N=3/sex) were treated with a single i.v. infusion administration at 10 mg/kg P4A1-2A in 60 mins infusion time. Blood samples collected at different timepoints and serum concentrations of P4A1-2A were determined with ELISA. Data are presented as mean ± SEM (N=3/sex). PK parameters derived are listed in the lower panel.

Supplementary Figure 9

a

Parameters examined

**Clinical observations**	1. Tolerance at injection sites
	2. Body weight
	3. Body weight change
	4. Qualitative food consumption
	5. Ophthalmology
	6. Body temperature
**Safety pharmacology**	1. Electrocardiography
	2. Blood pressure
	3. Respiration
	4. Neurological examination
**Ophthalmology**	
**Clinical pathology**	1. Hematology
	2. Serum chemistry
	3. Coagulation
	4. Urinalysis
**Toxicokinetics**	
**Immunogenicity**	Anti-drug-antibody (ADA)
**Cytokine**	IL-2, IL-4, IL-5, IL-6, IL-10, IL-13, TNF-α and IFN-γ
**Necropsy & Pathology**	1. Organ weight
	2. Gross evaluation
	3. Histopathology

- *Administration of P4A1-2A at 50 or 300 mg/kg/dose once per week for 2 weeks was well tolerated and did not result in any adverse changes.*
- *The no observed adverse effect level (NOAEL) for this study was considered to be 300 mg/kg/dose.*

b

Tissue cross-reactivity (TCR) with 37 types of frozen normal human tissues

Adrenal	Fallopian Tube	Spinal Cord
Bladder	Heart	Spleen
Blood cells	Kidney (glomerulus, tubule)	Stomach
Bone Marrow	Liver	Striated Muscle
Breast	Lung	Testis
Cerebellum	Lymph Note	Thymus
Cerebral Cortex	Ovary	Thyroid
Colon	Pancreas	Tonsil
Small Intestine	Pituitary	Ureter
Endothelium (blood vessel)	Placenta	Uterus (cervix)
Eye	Prostate	Uterus (endometrium)
Esophagus	Skin	-
Peripheral Nerve	Salivary Gland	-

- *No positive staining was observed in a panel of 37 human tissues (with each tissue in triplicates from 3 donors) when stained with 5 µg/mL or 25 µg/mL Biotin-P4A1-2A. All positive and negative controls worked as expected.*

Supplementary Fig.9. GLP-compliant safety evaluations: (a) Toxicology study; (b) Tissue cross-reactivity study.

Supplementary Figure 10

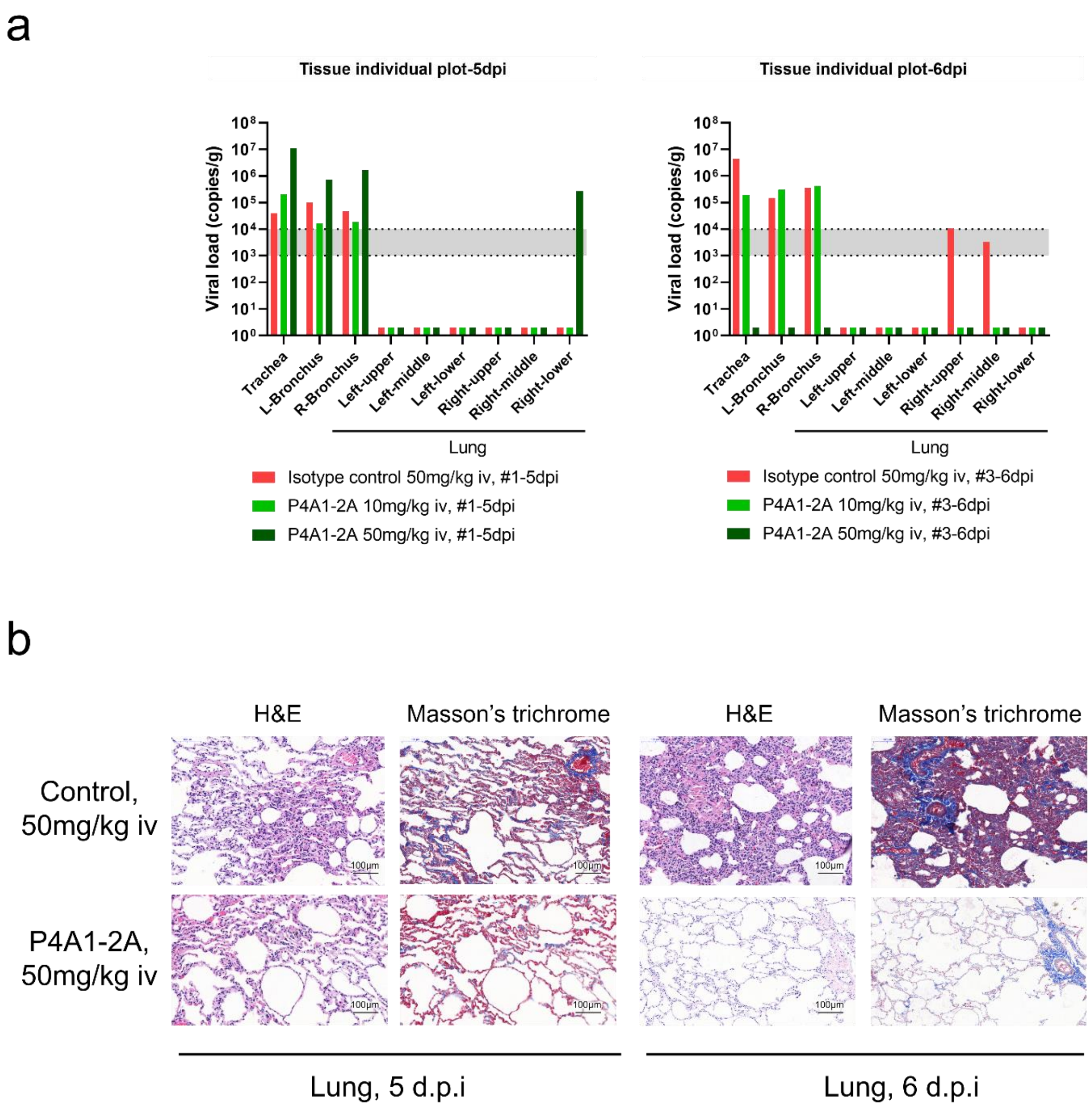

Supplementary Fig.10. Viral load and histopathology in lung tissue in the rhesus macaque model of SARS-CoV-2 infection 5 and 6 d.p.i. (a) Viral load in the respiratory tissues (including trachea, left and right bronchus, and all six lung lobes) collected at necropsy on 5 (left) or 6 (right) days post-infection (d.p.i.; n=1/group/day) were tested by RT-qPCR. (b) Representative images of histopathology in lung tissue from isotype control or P4A1-2A 50 mg/kg treated animals (collected at 5 and 6 d.p.i). Experiments were not repeated as controls were consistent with historical data and/or literature.

Supplementary Table 1. Data collection and refinement statistics.

Wavelength	0.97915
Resolution range	36.16 - 2.108 (2.183 - 2.108)
Space group	C 2 2 21
Unit cell	85.958 148.575 144.655 90 90 90
Total reflections	475973
Unique reflections	48014 (4316)
Multiplicity	9.6 (9.7)
Completeness (%)	89.21 (81.11)
Mean I/sigma(I)	23.5 (2.3)
Wilson B-factor	32.62
R-merge	0.091 (0.800)
R-meas	0.097 (0.844)
R-pim	0.031 (0.264)
CC1/2	0.996 (0.895)
Reflections used in refinement	47909 (4316)
Reflections used for R-free	1991 (185)
R-work	0.1858 (0.2309)
R-free	0.2284 (0.2616)
Number of non-hydrogen atoms	5466
macromolecules	4861
ligands	14
solvent	591
Protein residues	793
RMS(bonds)	0.007
RMS(angles)	0.95
Ramachandran favored (%)	97.1
Ramachandran allowed (%)	2.9
Ramachandran outliers (%)	0
Rotamer outliers (%)	0.55
Clashscore	4.8
Average B-factor	39.13
macromolecules	37.67
ligands	82.89
solvent	50.16
Number of TLS groups	1

Statistics for the highest-resolution shell are shown in parentheses.

Supplementary Table 2. Residues contributed to interaction between P4A1/SARS-CoV-2-RBD.

SARS-CoV-2 RBD	Distance (Å)			P4A1 Antibody
Hydrogen Bonds				
SER 477 [N]	3.1			VH:GLY 26 [O]
LYS 458 [NZ]	2.7			VH:SER 30 [O]
TYR 473 [OH]	2.8			VH:SER 31 [O]
TYR 449[OH]	4.0			VH:SER 31[OG]
LYS 458 [NZ]	3.1			VH:SER 53 [O]
TYR 421 [OH]	3.4			VH:SER 53 [OG]
LYS 417 [NZ]	2.7			VH:GLN 100 [OE1]
TYR 453 [OH]	2.4			VH:GLU 101 [OE1]
ASP 420 [OD2]	2.5			VH:SER 56 [OG]
TYR 421 [OH]	3.6			VH:SER 53 [N]
TYR 421 [OH]	2.9			VH:GLY 54 [N]
LEU 455 [O]	2.8			VH:TYR 33 [OH]
ARG 457 [O]	2.6			VH:SER 53 [OG]
ALA 475 [O]	3.2			VH:ILE 28 [N]
ALA 475 [O]	2.9			VH:ASN 32 [ND2]
ASN 487 [OD1]	2.9			VH:ARG 97 [NH1]
TYR 489 [OH]	3.4			VH:ARG 97 [NH2]
GLY 502 [N]	3.0			VL:GLY 28 [O]
ARG 403 [NH1]	2.9			VL:ASN 92 [O]
ARG 403 [NH2]	3.1			VL:ASN 92 [O]
TYR 505 [OH]	2.7			VL:SER 93 [OG]
ASN 501 [OD1]	3.2			VL:SER 30 [N]
GLY 496 [O]	3.0			VL:SER 30 [OG]
ASN 501 [OD1]	3.0			VL:SER 30 [OG]
GLN 498 [OE1]	3.4			VL:SER 67 [OG]
TYR 505 [OH]	3.6			VL:SER 93 [N]
Salt Bridge				
LYS 417 [NZ]	3.1			VH:GLU 101 [OE2]
Solvent Hydrogen Bond Bridge				
GLY 476 [O]	3.0	W740	3.0	VH: SER 31 [OG]
TYR 505 [OH]	2.8	W388	2.8	VL: TRP 32[O]
TYR 505 [OH]	2.8	W388	3.1	VL: ALA 91 [N]
TYR 505 [OH]	2.8	W388	2.9	VL: ASN 92 [N]

Supplementary Table 3. PISA analysis of interaction between P4A1/SARS-CoV-2-RBD

	Total surface area, Å^2		Interaction residues		Interface area, Å^2	ΔiG (kcal/m)	ΔiG (P-value)	N_{HB}	N_{SB}	N_{DS}	CSS
	RBD	HC/LC	RBD	HC/LC							
HC	10140	11747	25	23	780.7	-1.8	0.766	17	1	0	0.024
LC		11442	17	16	414.6	1.3	0.663	9	0	0	0.007

HC: Heavy chain; LC: Light Chain; ΔiG: Solvation free energy gain upon formation of the interface; N_{HB}: number of potential hydrogen bonds across the interface; N_{SB}: number of potential salt bridges across the interface; N_{DS}: number of potential disulfide bonds across the interface; CSS: Complexation Significance Score

Supplementary Table 4. Overview oligononucleotide primers used in this study

Purpose	Gene name/purpose	Primer	Sequences (5' -- 3')
**RBD expression**	SARS-CoV-2 RBD	Forward	TCTCCTACATCTACGCCGACGGATCCACCAACCTCTGCCCTTTCGGT
		Reverse	TGGTGATGGTGGTGATGATGTGCGGCCGCACTCTTCTTTGGCCCGCATA
**In vivo study viral load qPCR**	SARS-CoV-2 NP	Forward	GGGGAACTTCTCCTGCTAGAAT
		Reverse	CAGACATTTTGCTCTCAAGCTG
**Single B cell sequencing**	VH & VL library construction and sequencing	Forward	ACA GGT GCC CAC TCC CAG GTG CAG
			AAG GTG TCC AGT GTG ARG TGC AG
			CCC AGA TGG GTC CTG TCC CAG GTG CAG
			CAA GGA GTC TGT TCC GAG GTG CAG
			GCA GCA GCA ACA GGT GCC CAC
			GGC CTC CCA TGG GGT GTC CTG
			GCT GTT CTC CAA GGA GTC TGT
			GCA GCT CCC AGA TGG GTC CTG
			ATT TTA AGA GGT GTC CAG TGT
			ATT TTA AAA GGT GTC CAG TGT
			ATT TTA GAA GGT GTC CAG TGT
			ACC CCT TCC TGG GTC TTG TCC
			ATC CCT TCA TGG GTC TTG TCC
			GCA GCT ACA GGC ACC CAC GCC
			GCA GCC ACA AGT GCC CAC TCC
			GCA GCC ACA GGT GCC CAC TCC
			GCA GCC ACA GGA GCC CAC TCC
			GGT CCT GGG CCC AGT CTG TGC TG
			GGT CCT GGG CCC AGT CTG CCC TG
			GCT CTG TGA CCT CCT ATG AGC TG
			GGT CTC TCT CSC AGC YTG TGC TG
			GTT CTT GGG CCA ATT TTA TGC TG
			GGT CCA ATT CYC AGG CTG TGG TG

			GAG TGG ATT CTC AGA CTG TGG TG
			ACT CAC TCT GCA GTG TCA GTG GTC
			AGT CTC CTC ACA GGG TCC CTC TCC
			GCT TAT GGA TCA GGA GTG GAT TCT
			ACT TGC TGC CCA GGG TCC AAT TC
			CAC TGC ACA GGT TCT TGG GCC
			TCT CAC TGC ACA GGT TCC CTC TC
			CAC TGG ACA GGG TCT CTC TCC
			TTC TCC ACA GGT CTC TGT GCT
			CTC TAC ACA GGC TCT ATT GCC
			CTT TGC ATA GGT TCT GTG GTT
			TAC TGC ACA GGA TCC GTG GCC
			CTC TGC ACA GGC TCT GAG GCC
			TAC TGC ACA GGA TCC GTG GCC
			CAG GGC ACA GGA TCC TGG GCT
			CAG GGC ACA GGG TCC TGG GCC
			CAC TGC ACA GGG TCC TGG GCC
			CAC TGT GCA GGG TCC TGG GCC
			ATG AGG STC CCY GCT CAG CTG CTG G
			CTC TTC CTC CTG CTA CTC TGG CTC CCA G
			ATT TCT CTG TTG CTC TGG ATC TCT G
			TGG GTT CCA GCC TCC AGG GG
			TGG ATC TCT GAT ACC AGG GC
			TGG ATC TCT GGT GCC TAC GG
			TGG CTC CCA GAT ACC ACT GG
			TGG CTC CCA GAT ACC ACC GG
			CTC TGG GTC TCT GGA TCC AGT
			CTC TGG GTC CCA GGA TCC AGT
			CTC TGG GTC CCT GGA TCC AGT
			TGG CTC CCA GAT ACC AGA TGT
			TGG TTC CCA GGT TCC AGA TGC
			TGG CTC CCA GGT GCC AGA TGT

			TGG CTC TCA GGT GCC AGA TGT
			TGG CTC CGA GGT GCC AGA TGT
		Reverse	GCCAGGGGGAAGACCGATGG
			GAGGGCGGGAACAGAGTGAC
			CAACTGCTCATCAGATGGCG
