# Supplementary material for: A SARS-CoV-2 neutralizing antibody with extensive Spike binding coverage and modified for optimal therapeutic outcomes
Source: Nat Commun. 2021 May 11;12:2623. doi: 10.1038/s41467-021-22926-2 (PMC8113581; doi:10.1038/s41467-021-22926-2)
Supplement: Supplementary file 3 — Reporting Summary [file 41467_2021_22926_MOESM3_ESM.pdf]

nature research

Corresponding author(s): Yu Guo, Zihe Rao, Kun Sun, Liang Schweizer, Chao Shan, Hongkai Zhang, Fei Deng, Zhiyong Lou

Last updated by author(s): Mar 22, 2021

Reporting Summary

Nature Research wishes to improve the reproducibility of the work that we publish. This form provides structure for consistency and transparency in reporting. For further information on Nature Research policies, see our Editorial Policies and the Editorial Policy Checklist.

Statistics

For all statistical analyses, confirm that the following items are present in the figure legend, table legend, main text, or Methods section.

n/a	Confirmed	
☐	☒	The exact sample size (n) for each experimental group/condition, given as a discrete number and unit of measurement
☐	☒	A statement on whether measurements were taken from distinct samples or whether the same sample was measured repeatedly
☒	☐	The statistical test(s) used AND whether they are one- or two-sided<br>*Only common tests should be described solely by name; describe more complex techniques in the Methods section.*
☒	☐	A description of all covariates tested
☒	☐	A description of any assumptions or corrections, such as tests of normality and adjustment for multiple comparisons
☐	☒	A full description of the statistical parameters including central tendency (e.g. means) or other basic estimates (e.g. regression coefficient) AND variation (e.g. standard deviation) or associated estimates of uncertainty (e.g. confidence intervals)
☒	☐	For null hypothesis testing, the test statistic (e.g. F, t, r) with confidence intervals, effect sizes, degrees of freedom and P value noted<br>*Give P values as exact values whenever suitable.*
☒	☐	For Bayesian analysis, information on the choice of priors and Markov chain Monte Carlo settings
☒	☐	For hierarchical and complex designs, identification of the appropriate level for tests and full reporting of outcomes
☒	☐	Estimates of effect sizes (e.g. Cohen's d, Pearson's r), indicating how they were calculated

Our web collection on statistics for biologists contains articles on many of the points above.

Software and code

Policy information about availability of computer code

Data collection	Sequence data was collected by Illumina MiSeq PE300 approach with MiSeq Reagent Kit v3 and MiSeq system (catalog no. SY-410-1003)<br>Elisa data was collected with microplate reader (SpectraMax i3X suppled by Molecular Devices or MultiSkan supplied by ThermoFisher)<br>FACS data was collected with flow cytometry (CytoFlex, Beckman); Diffraction data were collected at the Shanghai Synchrotron Radiation Facility (SSRF) BL17U1 (wavelength, 0.97915 Å) at 100K.
Data analysis	Sequence data: using dedicated bioinformatics pipeline (AbsolutionTM) for analysis of single-cell antibody sequences developed by HiFiBiO Therapeutics as described in Gérard A et al. 2020. https://doi.org/10.1038/s41587-020-0466-7<br>FACS data was analyzed by Flowjo 10.6.0 and Graphpad Prism 8.0.1<br>Graphpad Prism 8.0.1 are used to analyze data, draw the curve and calculate EC50 .<br>BIAcore T200 Evaluation Software (version 3.1) are used to analysis of SPR data.<br>All data sets were processed using the HKL3000 package.<br>Structures were solved by molecular replacement using PHASER with the SARS-CoV-2 RBD structure (PDB ID: 6M0J)<br>The initial model was built into the modified experimental electron density using COOT(Versioin 0.9.4) and further refined in PHENIX(Version 1.19).<br>Model geometry was verified using the program MolProbity(Version 4.4). Structural figures were drawn using the program PyMOL(Version 1.8).<br>Epitope and paratope residues, as well as their interactions, were identified by accessing PISA incorporated in CCP4 Software Suite (version 7.1)

For manuscripts utilizing custom algorithms or software that are central to the research but not yet described in published literature, software must be made available to editors and reviewers. We strongly encourage code deposition in a community repository (e.g. GitHub). See the Nature Research guidelines for submitting code & software for further information.

Data

Policy information about availability of data

All manuscripts must include a data availability statement. This statement should provide the following information, where applicable:

- Accession codes, unique identifiers, or web links for publicly available datasets
- A list of figures that have associated raw data
- A description of any restrictions on data availability

The atomic models generated from X-ray crystallographic studies of the P4A1-RBD complex has been deposited at the Protein Data Bank (PDB, http://www.rcsb.org/) under accession codes PDB 7CJF [http://www.rcsb.org/structure/7CJF]. Source data are provided with this paper. The authors declare that all data are available within the article and its Supplementary Information files, or are available from the authors upon request.
All reagents and information presented in this study are available from corresponding authors upon reasonable request.

Field-specific reporting

Please select the one below that is the best fit for your research. If you are not sure, read the appropriate sections before making your selection.

[x] Life sciences [ ] Behavioural & social sciences [ ] Ecological, evolutionary & environmental sciences

For a reference copy of the document with all sections, see nature.com/documents/nr-reporting-summary-flat.pdf

Life sciences study design

All studies must disclose on these points even when the disclosure is negative.

Sample size	No statistical method was used to predetermine sample sizes.<br>To characterize the antiviral immune responses, antibody titers in 23 patient serums were determined by ELISA against the SARS-CoV-2 S protein. Because the amount of blood sample was limited, when we characterized the B cell populations of patient samples, PBMCs of two patients with the highest serum titer and one healthy donor were analyzed using flow cytometry.<br>Rhesus monkey COVID-19 model study: n=3/group was used based on predicted efficacy level and relevant literature.<br>Cynomolgus monkey PK study: n=3/sex was used based on routine practice, relevant literature and AAALAC 3R principal.<br>Cynomolgus monkey toxicology study: n=5/sex/group (dosing phase: n=3/sex/group; recovery phase: n=2/sex/group) was used based on routine practice, relevant literature and AAALAC 3R principal.
Data exclusions	No Data was excluded from the analysis.
Replication	Convalescent patient sample analyses: studies were repeated at least once with similar results.<br><br>Rhesus monkey efficacy study:<br>Oropharyngeal, nasal and rectal swabs, blood samples: harvested from all animals daily from 1-7 d.p.i. (if applicable) and viral load determined.<br>Viral titer in tissues: tissues were collected at necropsy at 5-7 d.p.i. from each group (n=1/day/group) for histopathology examination and viral load determination.<br>As for the viral load determination was repeated once with similar results. Histopathology evaluation was performed independently by investigator and a pathologist with similar findings.<br>Cynomolgus monkey PK study:<br>Blood samples were collected from each animal at each timepoint and blood testing antibody concentration was determined with a validated ELISA assay.<br>Cynomolgus monkey toxicology study:<br>Each animal was monitored daily with safety tests performed at least once pre-treatment, once after each of the 2 treatments, and once in the last week in recovery phase. Blood samples were collected from each animal at each timepoint and blood testing antibody or cytokine concentration was determined with validated ELISA assays.
Randomization	Randomization was not performed for the convalescent patients as 2 patients with highest titer were selected for the study; Randomization was performed for the rhesus monkey efficacy, cynomolgus monkey toxicology studies. Randomization was not performed for cynomolgus monkey PK study as only one dose group was tested, but the animals were randomly selected for enrollment.
Blinding	Researchers of the study used internal patient number during the study, only have access to basic demographic information of patient including gender, age, as well as time of diagnosis, recovery and severity of the disease. Researchers were blinded on all personal information that can be used to identify the patient. Sample testing was performed without knowing any patient information associated.<br>The investigators were not blinded to group allocation during the collection of specimens from animal (rhesus and cynomolgus monkeys), sample testing was performed by independent laboratories with code used to identify sample and timepoints.

Reporting for specific materials, systems and methods

We require information from authors about some types of materials, experimental systems and methods used in many studies. Here, indicate whether each material, system or method listed is relevant to your study. If you are not sure if a list item applies to your research, read the appropriate section before selecting a response.

Materials & experimental systems

n/a	Involved in the study	
☐	☒	Antibodies
☐	☒	Eukaryotic cell lines
☒	☐	Palaeontology and archaeology
☐	☒	Animals and other organisms
☐	☒	Human research participants
☒	☐	Clinical data
☒	☐	Dual use research of concern

Methods

n/a	Involved in the study	
☒	☐	ChIP-seq
☐	☒	Flow cytometry
☒	☐	MRI-based neuroimaging

Antibodies

Antibodies used

For binding ELISA:
- Peroxydase Affinipure F(ab')2 goat anti-human IgG (H+L); Jackson ImmunoResearch, Cat # 109-036-088; 1:10,000
- Sheep anti-human C1q Ab-HRP (Complement Technology; Cat # CPBT-65026SH; 1:300)

Flow cytometry: dilutions 1:70
- anti-CD19 (FITC labeled, eBioscience, catalog # 11-0199-42, clone name: HIB19)
- anti-CD27 (APC labeled, eBiosciences, catalog # 17-0279-42, clone name: O323)
- anti-CD38 (PE labeled, eBioscience, catalog # 12-0388-42, clone name: HB7)
- Isotype PE Mouse isotype control (BioLegend, catalog # 400114, clone name: MOPC-21)
- FITC labeled mouse IgG1 isotype control (ebioscience, catalog # 11-4714-41, clone name: P3.6.2.8.1)
- APC labeled mouse IgG1 isotype control (BD Biosciences, catalog#550854, clone name: MOPC-21)
- Rabbit anti-mouse IgG Fc-AF647 (Jackson ImmunoResearch, catalog # 315-606-046, 1:800 dilution)

Live virus neutralization study:
- anti-SARS-CoV-2 nucleocapsid protein (NP) rabbit serum, produced internally, 1:1000 dilution
- Goat Anti-Rabbit IgG H&L (Alexa Fluor® 488) (1:500 dilution, Abcam, Cambridge, UK; ab150077; Lot#: GR3244688-2)

Validation

The anti-SARS-CoV-2 NP rabbit serum was prepared and used as primary antibody for the IFA tests. Rabbit was inoculated with purified NP expressed in E.coli for three times. Serum was harvested and was validated by Western Blot using NP transfected HEK293 cells and SARS-CoV-2 infected Vero E6 cells .
The other antibodies in our study are all commercial available and their specificity are well characterized by the manufactors and other users.

All antibodies were validated and dilution optimized using positive cells (antigen-transfected 293T cells or PBMC) before performance of the study. The information are included in the Methods section.

Eukaryotic cell lines

Policy information about cell lines

Cell line source(s)

Flow cytometry testing block of S1-ACE2 binding:
- Vero-E6 cell line: ATCC, CRL-1587

Pseudovirus testing:
- Huh-7 cells, acquired from ATCC.
- HEK293 cells, acquired from ATCC.

Live virus neutralization assay:
- Vero E6 cell line: ATCC; CRL-1586, Lot#: 60526234

Antibody verification:
- 293T cells: acquired from Chinese Academy of Sciences; cat# GNHu17

Protein production
- Initial expression: ExpiCHO cell: Gibco, No. A29133.
- P4A1-2A: CHO.K1 cells: originally from ATCC, No. CCL 61.

Authentication

Flow cytometry testing block of S1-ACE2 binding:
- Vero-E6 cell line validated by CoBIOER (http://www.cobioer.com/)

Pseudovirus testing:
- Huh-7 cells, not authenticated.
- HEK293 cells, not authenticated.

Live virus neutralization assay:
- Vero E6 cell line: not authenticated)

Antibody verification:
- 293T cells: authenticated by STR analysis

Protein production

	• Initial expression: ExpiCHO cell: not authenticated.<br>• P4A1-2A: CHO.K1 cells, qualified for use based on full characterization on identity, morphology, and freedom from adventitious agents
Mycoplasma contamination	All cells are tested negative for mycoplasma contamination.
Commonly misidentified lines (See ICLAC register)	No commonly misidentified cell lines were used in the study.

Animals and other organisms

Policy information about studies involving animals; ARRIVE guidelines recommended for reporting animal research

Laboratory animals	Efficacy study: 9 rhesus monkeys (three males, six females; 6–7 years of age, 5.3-7.3 kg) was randomized into 3 treatment groups (n= 1 male and 2 females) and received a single iv treatment of isotype control 50 mg/kg or P4A1-2A at 10 or 50 mg/kg 1 day after intratracheal viral challenge.<br>PK study: 6 naïve cynomolgus monkeys (3/sex) were randomly selected and enrolled in the study and receive a single iv treatment of P4A1-2A at 10mg/kg.<br>Toxicology study: 30 cynomolgus monkeys (15/sex, 3-5 years old; 2.2 to 3.6 kg for females and 2.3 to 5.2 kg for males) were randomly assigned to 3 groups of 5/sex/group and received once weekly treatment of placebo, P4A1-2A at 50 or 300 mg/kg/dose for 2 weeks.
Wild animals	This study did not involve wild animals
Field-collected samples	This study did not involve field-collected samples.
Ethics oversight	All animal experiments were performed following Association for Assessment & Accreditation of Lab Animal Care International (AAALAC) guidelines and all relevant ethical regulations. The study protocol was approved by the Institutional Animal Care and Use Committee (IACUC) of Wuhan Institute of Virology, Chinese Academy of Sciences (Ethics number: WIVA42202001) or Wuxi AppTec (Number: SZ20200529-Monkeys for PK study and SZ20200608-Monkeys for GLP Toxicology study). Rhesus macaque studies were conducted within the animal biosafety level 4 (ABSL- 4) facility in the National Biosafety Laboratory (Wuhan), Chinese Academy of Sciences.

Note that full information on the approval of the study protocol must also be provided in the manuscript.

Human research participants

Policy information about studies involving human research participants

Population characteristics	Total of 23 patients were recruited from Jan 11rd, 2020 to March 1st, 2020. There are 13 males. The medium age is 47.0 (Q1-Q3: 31.0-51.0) years old. Most of them had symptoms of fever and cough at diagnosis and admission to the hospital, but recovered (no symptoms and PCR negative) and were released from hospital at the time of sample collection.
Recruitment	Convalescent COVID-19 patients were recruited from recovered patients, who attended scheduled follow-up visits after hospital discharge between February 11th, 2020 and March 1st, 2020 at Yongjia People's Hospital and Yongjia Center for Disease Control and Prevention in Zhejiang Province, China. The participants expressed willingness to participate in the study and signed Informed Consent Form.<br>2 patients with the highest antibody titer were selected for the study.
Ethics oversight	The study protocol was approved by the Ethics Committee of Xinhua Hospital affiliated to Shanghai Jiao Tong University (approval #XHEC-C-2020-006-2), Yongjia People's Hospital and Yongjia Center for Disease Control and Prevention in Zhejiang Province. Blood samples were collected from convalescent COVID-19 patients and healthy volunteers with signed informed consent forms according to study protocol approved by IRBs.

Note that full information on the approval of the study protocol must also be provided in the manuscript.

Flow Cytometry

Plots

Confirm that:

- [x] The axis labels state the marker and fluorochrome used (e.g. CD4-FITC).
- [x] The axis scales are clearly visible. Include numbers along axes only for bottom left plot of group (a 'group' is an analysis of identical markers).
- [ ] All plots are contour plots with outliers or pseudocolor plots.
- [x] A numerical value for number of cells or percentage (with statistics) is provided.

Methodology

Sample preparation	PBMC was thawed at 37°C and then centrifuged at 450 g for 8 min. The supernatant was discarded, and the cells resuspended in 200 μL of DMEM. Following the addition of 1μL of Dnase I cells were incubated for 3 min and spun down again. The pellet was resuspended in 20μL of FcR Blocking Reagent, incubated for 10 mins and centrifuged. The cells were suspended in 200μL PBS. 3uL of CD19 (FITC labeled, eBioscience 11-0199-42), CD27 (APC labeled, eBiosciences 17-0279-42),

anti CD38 (PE labeled mouse IgG1 isotype control, eBioscience 12-0388-42) or its isotype (PE Mouse isotype control, BioLegend 400114 and FITC labeled mouse IgG1 isotype control, ebioscience 11-4714-41 and APC labeled mouse IgG1 isotype control, BD 550854) was then added, and incubated for 30 min at room temperature. Following centrifugation, cells were resuspended in a 100µL of 4% PFA. After 10 min the cells were washed twice by centrifugation and finally resuspended in PBS and ready for flow cytometry analysis using a Cytoflex, Beckman Coulter. The median fluorescence intensity (MFI) was calculated with FlowJo.

Instrument	Cytoflex, Beckman Coulter
Software	Flowjo
Cell population abundance	15000 events were recorded and a series of hierachical gates were applied to isolate the target cells. 10000 cells were gating within lymphocytes population. CD19 positive cells (5%~9%) were gated within lymphocytes population.2% plasma cells were gated within CD19 positive cells; 20%~40% memory B cells were gated within CD19 positive cells.
Gating strategy	lymphocytes population was gated using FSC/SSC dot plot, then CD19 positive cells were gated within CD19 FITC-A/SSC-A under lymphocytes population; CD27 and CD38 staining within the CD19+ cell population. plasma cells (2%) were gating within the CD27+/CD38+/CD19+; Memory B cells(20%~40%) were gating within the CD27+/CD38-/CD19+.

☒ Tick this box to confirm that a figure exemplifying the gating strategy is provided in the Supplementary Information.
